# Enhanced Negative Nonlocal Conductance in an Interacting Quantum Dot Connected to Two Ferromagnetic Leads and One Superconducting Lead

**DOI:** 10.3390/e21101003

**Published:** 2019-10-14

**Authors:** Cong Lee, Bing Dong, Xiao-Lin Lei

**Affiliations:** Key Laboratory of Artificial Structures and Quantum Control (Ministry of Education), Department of Physics and Astronomy, Shanghai Jiaotong University, 800 Dongchuan Road, Shanghai 200240, China; kitozumika@sina.cn (C.L.); xllei@sjtu.edu.cn (X.-L.L.)

**Keywords:** superconducting proximity effect, Kondo effect, spin polarization, Anreev reflection

## Abstract

In this paper, we investigate the electronic transport properties of a quantum dot (QD) connected to two ferromagnetic leads and one superconducting lead in the Kondo regime by means of the finite-*U* slave boson mean field approach and the nonequilibrium Green function technique. In this three-terminal hybrid nanodevice, we focus our attention on the joint effects of the Kondo correlation, superconducting proximity pairing, and spin polarization of leads. It is found that the superconducting proximity effect will suppress the linear local conductance (LLC) stemming from the weakened Kondo peak, and when its coupling $\Gamma_{s}$ is bigger than the tunnel-coupling Γ of two normal leads, the linear cross conductance (LCC) becomes negative in the Kondo region. Regarding the antiparallel configuration, increasing spin polarization further suppresses LLC but enhances LCC, i.e., causing larger negative values of LCC, since it is beneficial for the emergence of cross Andreev reflection. On the contrary, for the parallel configuration, with increasing spin polarization, the LLC decreases and greatly widens with the appearance of shoulders, and eventually splits into four peaks, while the LCC decreases relatively rapidly to the normal conductance.

## 1. Introduction

Recently, electron transport through a hybrid nanodevice, for instance, a quantum dot (QD), connected to normal and superconducting electrodes, has attracted much attention in many experimental [1,2,3,4,5,6,7,8,9,10,11,12,13,14,15,16,17,18,19,20] and theoretical studies [21,22,23,24,25,26,27,28,29] due to the associated physical challenges and potential applications in spintronics and quantum information. When a QD is connected to a superconductor, superconducting order can leak into it to give rise to pairing correlations and an induced superconducting gap, known as the superconducting proximity effect; this privileges the tunneling of Cooper pairs of electrons with opposite spin, and thereby favors QD states with even numbers of electrons and a zero total spin. At the same time, the local Coulomb repulsion enforces a one-by-one filling of the QD, and thereby induces the Coulomb blockade and even the Kondo effect at very low temperatures, which exhibits the zero-bias anomaly in the differential conductance with odd numbers of electrons residing in the QD. In this case, the superconducting proximity effect competes with the on-site Coulomb correlation [1,6,10,21,24,25,28,29].

It is even more intriguing when the QD additionally connects to a ferromagnetic lead [30,31]. It is known that the effective exchange field induced by the ferromagnetic correlation can cause a spin imbalance inside the QD, and as a result, suppress and/or even split the Kondo peak in the differential conductance [32,33,34,35,36,37]. Furthermore, spin polarization of the QD, on the one hand, is disadvantageous to the formation of on-dot superconducting pairing. However, the spin polarization in the antiparallel configuration, on the other hand, is favorable to the Andreev reflection (AR) and Cooper pair splitting [30,38]. It is, therefore, very interesting to study how the interplay of the Kondo, superconducting pairing, and ferromagnetic correlations affects the electron tunneling through a QD [39]. In a recent paper, Futterer et al. present a theoretical analysis of the subgap transport of such a three-terminal hybrid system, which consists of n interacting QD attached to two ferromagnetic leads and one superconducting lead [40,41]. They focused on the first-order sequential tunneling by using a master equation and found that the strong on-dot electron–electron interaction, rather than the nonlocal AR, leads to negative values of the nonlocal current response at an appropriately large bias voltage. Moreover, the bias-dependent supercurrent in the superconducting electrode was proposed as a sensitive detector to probe the exchange field of the QD induced by ferromagnetic leads [42]. Thereafter, the tunneling magnetoresistance was calculated for the same system to display a nontrivial dependence on the bias voltage and the level detuning caused by the AR [43]. Very recently, it has been reported, in contrast to [40], that the cross AR is indeed the dominant nonlocal transport channel at a low bias voltage and leads to a negative value of the cross conductance in the three-terminal hybrid nanodevice with two normal electrodes instead [44,45].

In the present work, we extend the finite-*U* slave boson mean field (SBMF) approach of Kotliar and Ruckenstein [46] with the help of the nonequilibrium Green function (NGF) method to investigate the subgap transport for the same three-terminal hybrid QD as in [40]. This kind of SBMF approach is generally believed to be reliable in describing not only spin fluctuations rigorously but also charge fluctuations to a certain degree in the Kondo regime at zero temperature [46,47,48,49]. This nonperturbative approach has been successfully utilized to calculate the linear and nonlinear conductance within a relatively wide dot-level range from the mixed valence to the empty orbital regimes, in which the major characteristics induced by the external magnetic field and the magnetization in Kondo transport arise [49,50,51,52]. Furthermore, this approach has been applied to analyze the π-phase transition in a double-QDs Josephson junction caused by competition between Kondo and interdot antiferromagnetic coupling [53]. The main purpose of this paper is to analyze in detail the interplay of the Kondo, superconducting proximity induced on-dot pairing, and ferromagnetic correlations and their influence on electronic tunneling.

The rest of the paper is organized as follows. In Section 2, we introduce our model of the three-terminal hybrid system, and the equivalent slave-boson field Hamiltonian. Then, we present the self-consistent equations of the expectation values of slave-boson operators within the SBMF approach and NGF method. Moreover, the formulas for current and linear conductance, including the local and cross conductances, are given. In Section 3, we present and analyze our numerical calculations for the linear conductance and nonlinear conductance in detail. Finally, a brief summary is given in Section 4.

## 2. Model and Theoretical Formulation

### 2.1. Model Hamiltonian

**Figure 1:**
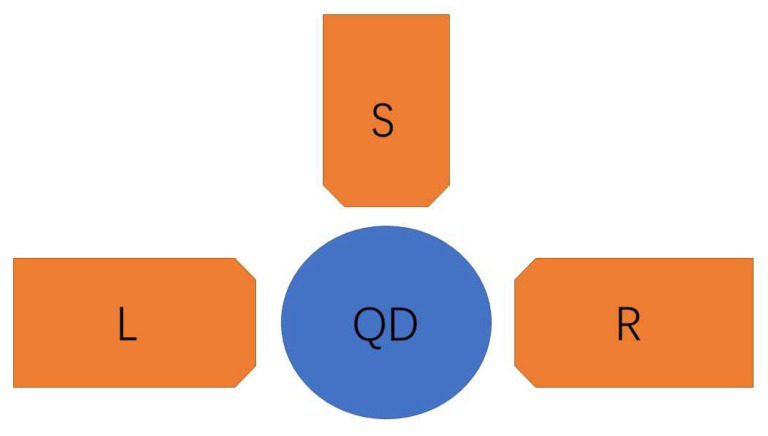
(Color online) Schematic diagram of a quantum dot connected to one superconducting lead and two ferromagnetic leads.

We consider a three-terminal hybrid nanodevice: an interaction QD connected to one superconducting lead and two ferromagnetic leads, as shown in Figure 1. The Hamiltonian of the system can be written as [40]

(1) $$H=H_{L}+H_{R}+H_{QD}+H_{T},$$

where

(2) $$H_{\eta }=\sum_{k\sigma }\epsilon_{\eta k\sigma }c_{\eta k\sigma }^{†}c_{\eta k\sigma },$$

(3) $$H_{QD}=\sum_{\sigma }\epsilon_{d}c_{d\sigma }^{†}c_{d\sigma }+Un_{1}n_{2}+\Gamma_{s}\left(c_{d1}^{†}c_{d2}^{†}+c_{d1}c_{d2}\right),$$

(4) $$H_{T}=\sum_{\eta k\sigma }\left(V_{\eta k}c_{\eta k\sigma }^{†}c_{d\sigma }+H.c.\right).$$

Here, η=L,R denotes the left and right leads, while σ=1,2 represents the spin degree of freedom. In the above equations, $c_{\eta k\sigma }^{†}$ ($c_{\eta k\sigma }$) and $c_{d\sigma }^{†}$ ($c_{d\sigma }$) are creation (annihilation) operators of electrons with spin σ in the η-th ferromagnetic lead and in the QD, respectively. In the dot Hamiltonian $H_{QD}$, $\epsilon_{d}$ is the energy level of the QD, $n_{\sigma }=c_{d\sigma }^{†}c_{d\sigma }$, and *U* is the on-site Coulomb repulsion between opposite spin electrons. $H_{T}$ depicts the tunneling between the QD and the two ferromagnetic leads, and $V_{\eta k}$ is the corresponding tunneling matrix element. In general, the tunneling amplitude $V_{\eta k}$ is assumed to be independent of spin and energy, and thus the effect of spin-polarized tunneling is captured by the spin-dependent tunneling rates, $\Gamma_{\eta \sigma }=2\pi \sum_{k}{\left|V_{\eta k}\right|}^{2}\delta \left(\omega -\epsilon_{\eta k\sigma }\right)$.

In this paper, since we are only interested in the subgap tunneling, it is natural to consider the limit of an extremely large superconducting gap in the superconducting lead. Therefore, the degree of freedom of the superconducting lead can be integrated out and an effective term can be constructed in the dot Hamiltonian, the third term in Equation (3). The parameter $\Gamma_{s}$ plays the role of describing the superconducting proximity effect on the dot. It is evident that this new proximized term mixes the empty state |0〉 and the doubly occupied state |↑↓〉 in the dot, and results in two new eigenstates with energies, $E_{\pm }=\varepsilon \pm \sqrt{\varepsilon^{2}+\Gamma_{s}^{2}}$ (here $\varepsilon =\epsilon_{d}+U/2$), which are known as the Andreev bound states. What we are interested in this paper is the effect of Andreev reflection on the electron tunneling through an interacting QD in the Kondo regime.

According to the finite-*U* slave-boson approach, one can introduce four additional auxiliary boson operators, *e*, $p_{\sigma }$, and *d*, which are associated with the empty, singly occupied, and doubly occupied electron states, respectively, of the QD, to discuss the above problem without interparticle couplings in an enlarged space with constraints: The completeness relation [46]

(5) $$\sum_{\sigma }p_{\sigma }^{†}p_{\sigma }+e^{†}e+d^{†}d=1,$$

and the particle number conservation condition

(6) $$c_{d\sigma }^{†}c_{d\sigma }=p_{\sigma }^{†}p_{\sigma }+d^{†}d.$$

Within the mean-field scheme, the effective Hamiltonian becomes (please see Appendix A) [46]

(7) $$\begin{array}{cc} H= & \sum_{\sigma }\epsilon_{d}c_{d\sigma }^{†}c_{d\sigma }+Ud^{†}d+\Gamma_{s}\left(z_{1}^{*}z_{2}^{*}c_{d1}^{†}c_{d2}^{†}+z_{1}z_{2}c_{d1}c_{d2}\right)+\sum_{\eta k\sigma }\left(V_{\eta k}c_{\eta k\sigma }^{†}c_{d\sigma }z_{\sigma }+V_{\eta k}^{*}c_{d\sigma }^{†}c_{\eta k\sigma }z_{\sigma }^{*}\right)\\ \; & +\sum_{\eta k\sigma }\epsilon_{\eta k\sigma }c_{\eta k\sigma }^{†}c_{\eta k\sigma }+\lambda^{1}\left(\sum_{\sigma }p_{\sigma }^{†}p_{\sigma }+e^{†}e+d^{†}d-1\right)+\sum_{\sigma }\lambda_{\sigma }^{2}\left(c_{d\sigma }^{†}c_{d\sigma }-p_{\sigma }^{†}p_{\sigma }-d^{†}d\right), \end{array}$$

where three Lagrange multipliers $\lambda^{1}$ and $\lambda_{\sigma }^{2}$ are drawn in order to make the constraints valid, and $z_{\sigma }$ is the correctional parameters in the hopping term to recover the many-body effect on tunneling with

(8) $$z_{\sigma }={\left(1-d^{†}d-p_{\sigma }^{†}p_{\sigma }\right)}^{-1/2}\left(e^{†}p_{\sigma }+p_{\bar{\sigma }}^{†}d\right){\left(1-e^{†}e-p_{\sigma }^{†}p_{\sigma }\right)}^{-1/2}.$$

### 2.2. Self-Consistent Equations

From the effective Hamiltonian Equation (7), one can derive four equations of the motion of slave-boson operators, which serve as the basic equations together with the three constraints. Then, we further apply the mean-field approximation in the statistical expectations of these equations, where all the boson operators are replaced by their respective expectation values. After a lengthy and tedious calculation employing the Langreth technique (please see the Appendix B for the details of derivation), we can obtain the self-consistent equations as follows [49,50,51,52]:

(9) $$\Gamma_{s}\frac{\partial (z_{1}z_{2})}{\partial e}\left(R+R^{*}\right)+\lambda^{1}e+\sum_{\sigma }\frac{\partial z_{\sigma }}{\partial e}\left(Q_{\sigma }+Q_{\sigma }^{*}\right)=0,$$

(10) $$\Gamma_{s}\frac{\partial (z_{1}z_{2})}{\partial p_{1}}\left(R+R^{*}\right)+\left(\lambda^{1}-\lambda_{1}^{2}\right)p_{1}+\frac{\partial z_{1}}{\partial p_{1}}\left(Q_{1}+Q_{1}^{*}\right)+\frac{\partial z_{2}}{\partial p_{1}}\left(Q_{2}+Q_{2}^{*}\right)=0,$$

(11) $$\Gamma_{s}\frac{\partial (z_{1}z_{2})}{\partial p_{2}}\left(R+R^{*}\right)+\left(\lambda^{1}-\lambda_{2}^{2}\right)p_{2}+\frac{\partial z_{1}}{\partial p_{2}}\left(Q_{1}+Q_{1}^{*}\right)+\frac{\partial z_{2}}{\partial p_{2}}\left(Q_{2}+Q_{2}^{*}\right)=0,$$

(12) $$\Gamma_{s}\frac{\partial (z_{1}z_{2})}{\partial d}\left(R+R^{*}\right)+\left(U+\lambda^{1}-\sum_{\sigma }\lambda_{\sigma }^{2}\right)d+\sum_{\sigma }\frac{\partial z_{\sigma }}{\partial d}\left(Q_{\sigma }+Q_{\sigma }^{*}\right)=0,$$

(13) $$\sum_{\sigma }|p_{\sigma }{|}^{2}+{\left|e\right|}^{2}+{\left|d\right|}^{2}-1=0,$$

(14) $$K_{\sigma }-|p_{\sigma }{|}^{2}-{\left|d\right|}^{2}=0,$$

where

(15) $$K_{1}=\langle c_{d1}^{†}c_{d1}\rangle =\int \frac{d\omega }{2\pi i}{\langle \langle c_{d1};c_{d1}^{†}\rangle \rangle }^{<}\left(\omega \right)=\frac{1}{2\pi i}\int d\omega G_{d11}^{<}\left(\omega \right),$$

(16) $$K_{2}=\langle c_{d2}^{†}c_{d2}\rangle =\int \frac{-d\omega }{2\pi i}{\langle \langle c_{d2}^{†};c_{d2}\rangle \rangle }^{>}\left(\omega \right)=\frac{-1}{2\pi i}\int d\omega G_{d22}^{>}\left(\omega \right),$$

(17) $$R=\langle c_{d1}^{†}c_{d2}^{†}\rangle =\int \frac{d\omega }{2\pi i}{\langle \langle c_{d2}^{†};c_{d1}^{†}\rangle \rangle }^{<}\left(\omega \right)=\frac{1}{2\pi i}\int d\omega G_{d21}^{<}\left(\omega \right),$$

(18) $$\begin{array}{cc} Q_{1\eta }= & z_{1}\Gamma_{\eta 1}\int \frac{d\omega }{2\pi }\left\{-\frac{i}{2}\left[{\tilde{\Gamma }}_{L1}f_{L}\left(\omega \right)+{\tilde{\Gamma }}_{R1}f_{R}\left(\omega \right)\right]{\left|G_{d11}^{R}\left(\omega \right)\right|}^{2}\right.\\ \; & \left.-\frac{i}{2}\left[{\tilde{\Gamma }}_{L2}\left(1-f_{L}\left(-\omega \right)\right)+{\tilde{\Gamma }}_{R1}\left(1-f_{R}\left(-\omega \right)\right)\right]{\left|G_{d21}^{R}\left(\omega \right)\right|}^{2}+f_{\eta }\left(\omega \right)G_{d11}^{A}\left(\omega \right)\right\}, \end{array}$$

(19) $$\begin{array}{cc} Q_{2\eta }= & z_{2}\Gamma_{\eta 2}\int \frac{d\omega }{2\pi }\left\{\frac{i}{2}\left[{\tilde{\Gamma }}_{L1}\left(1-f_{L}\left(\omega \right)\right)+{\tilde{\Gamma }}_{R1}\left(1-f_{R}\left(\omega \right)\right)\right]{\left|G_{d21}^{R}\left(\omega \right)\right|}^{2}\right.\\ \; & \left.+\frac{i}{2}\left[{\tilde{\Gamma }}_{L2}f_{L}\left(-\omega \right)+{\tilde{\Gamma }}_{R1}f_{R}\left(-\omega \right)\right]{\left|G_{d22}^{R}\left(\omega \right)\right|}^{2}-f_{\eta }\left(-\omega \right)G_{d22}^{A}\left(\omega \right)\right\}, \end{array}$$

and

(20) $$Q_{\sigma }=\sum_{\eta }Q_{\sigma \eta }.$$

Here, the QD Keldysh NGFs, $G_{d\sigma \sigma^{\prime }}^{R(A,<,>)}\left(\omega \right)$, are the matrix elements of the 2×2 retarded (advanced and correlation) GF matrix $G_{d}^{R(A,<,>)}\left(\omega \right)={\langle \langle \phi ;\phi^{†}\rangle \rangle }^{R(A,<,>)}$ defined in the Nambu presentation, in which the mixture Fermion operator, $\phi ={\left(c_{d1},c_{d2}^{†}\right)}^{T}$, has to be introduced to describe the electronic dynamics due to the superconducting proximity effect. For the effective noninteracting Hamiltonian, the retarded and advanced GFs $G_{d}^{R(A)}$ can be easily written in the frequency domain as

(21) $${\left(G_{d}^{R(A)}\left(\omega \right)\right)}^{-1}=\left[\begin{array}{cc} \omega -\epsilon_{d}-\lambda_{1}^{2}\pm \frac{i}{2}\left({\tilde{\Gamma }}_{L1}+{\tilde{\Gamma }}_{R1}\right) & -\Gamma_{s}z_{1}z_{2}\\ -\Gamma_{s}z_{1}^{*}z_{2}^{*} & \omega +\epsilon_{d}+\lambda_{2}^{2}\pm \frac{i}{2}\left({\tilde{\Gamma }}_{L2}+{\tilde{\Gamma }}_{R2}\right) \end{array}\right],$$

with the renormalized parameters, ${\tilde{\Gamma }}_{\eta \sigma }={\left|z_{\sigma }\right|}^{2}\Gamma_{\eta \sigma }$. In addition, the correlation GFs $G_{d}^{<(>)}\left(\omega \right)$ can be obtained with the help of the following Keldysh relation typical for a noninteracting system:

(22) $$G_{d}^{<(>)}\left(\omega \right)=G_{d}^{R}\left(\omega \right)\left[\Sigma_{L}^{<(>)}\left(\omega \right)+\Sigma_{R}^{<(>)}\left(\omega \right)\right]G_{d}^{A}\left(\omega \right),$$

with the self-energies

(23) $$\Sigma_{\eta }^{<}\left(\omega \right)=i\left[\begin{array}{cc} {\tilde{\Gamma }}_{\eta 1}f_{\eta }\left(\omega \right) & 0\\ 0 & {\tilde{\Gamma }}_{\eta 2}\left[1-f_{\eta }\left(-\omega \right)\right] \end{array}\right],$$

and

(24) $$\Sigma_{\eta }^{>}\left(\omega \right)=-i\left[\begin{array}{cc} {\tilde{\Gamma }}_{\eta 1}\left[1-f_{\eta }\left(\omega \right)\right] & 0\\ 0 & {\tilde{\Gamma }}_{\eta 2}f_{\eta }\left(-\omega \right) \end{array}\right],$$

where $f_{\eta }\left(\omega \right)=1/\left(e^{\beta (\omega -\mu_{\eta })}+1\right)$ is the Fermi distribution function of the lead η with the chemical potential $\mu_{\eta }$ and temperature 1/β.

### 2.3. The Current and Linear Conductance

The electric current flowing from the lead η into the QD can be obtained from the rate of change of the electron number operator of the left lead:

(25) $$I_{\eta }=\sum_{\sigma }I_{\eta \sigma }=-e\sum_{\sigma }\langle \frac{d}{dt}\sum_{k}c_{\eta k\sigma }^{†}c_{\eta k\sigma }\rangle .$$

After standard calculation, the current for the left lead can be written as [44,45]

(26) $$I_{L}=I_{L}^{ET}+I_{L}^{DAR}+I_{L}^{CAR},$$

with

(27) $$I_{L}^{ET}=\frac{e}{h}\int d\omega \left\{{\tilde{\Gamma }}_{L1}{\tilde{\Gamma }}_{R1}\left[f_{L}\left(\omega \right)-f_{R}\left(\omega \right)\right]{\left|G_{d11}^{R}\left(\omega \right)\right|}^{2}\right.\left.+{\tilde{\Gamma }}_{L2}{\tilde{\Gamma }}_{R2}\left[f_{L}\left(-\omega \right)-f_{R}\left(-\omega \right)\right]{\left|G_{d22}^{R}\left(\omega \right)\right|}^{2}\right\},$$

(28) $$I_{L}^{DAR}=\frac{2e}{h}\int d\omega {\tilde{\Gamma }}_{L1}{\tilde{\Gamma }}_{L2}\left[f_{L}\left(\omega \right)+f_{L}\left(-\omega \right)-1\right]\times {\left|G_{d12}^{R}\left(\omega \right)\right|}^{2},$$

(29) $$I_{L}^{CAR}=\frac{e}{h}\int d\omega \left\{{\tilde{\Gamma }}_{L1}{\tilde{\Gamma }}_{R2}\left[f_{L}\left(\omega \right)+f_{R}\left(-\omega \right)-1\right]\right.\left.+{\tilde{\Gamma }}_{L2}{\tilde{\Gamma }}_{R1}\left[f_{L}\left(-\omega \right)+f_{R}\left(\omega \right)-1\right]\right\}{\left|G_{d12}^{R}\left(\omega \right)\right|}^{2}.$$

The corresponding currents for the right lead can be readily obtained by simply exchanging the subscripts L and R in Equations (27)–(29). It is found that the current can be divided into three parts: $I_{L}^{ET}$ describes the single-particle tunneling current caused by the normal electron transfer (ET) processes from the left lead directly to the right lead; $I_{L}^{DAR}$ denotes the local Andreev current caused by the direct AR (DAR) processes in which an electron injecting from the left lead forms a Cooper pair in the superconducting lead, and at the same time, is reflected as a hole back into the left lead; and $I_{L}^{CAR}$ is the nonlocal Andreev current caused by the crossed AR (CAR) processes, which is similar to DAR except that the hole is reflected into another lead, i.e., here, the right lead.

Since we are interested in the interplay between the Andreev bound state and the Kondo effect in the nonlocal subgap tunneling, we choose the bias voltage configuration in this hybrid three-terminal nanodevice as follows: The left lead is biased with the chemical potential *V*, while the right lead and the superconducting electrode are both in contact with the ground. Therefore, one can define two different linear conductances: The usual local conductance $G_{L}=\partial I_{L}{/\partial V|}_{V=0}$ and the unusual nonlocal (cross) conductance $G_{C}=\partial I_{R}{/\partial V|}_{V=0}$, which is related to the nonlocal current response of the hybrid three-terminal nanodevice to external driving field, i.e., current flowing in the right lead caused by the bias voltage applied to the left lead. From Equations (27)–(29), the local conductance reads

(30) $$G_{L}=\frac{\partial I_{L}}{\partial V}{|}_{V=0}=G^{ET}+G^{DAR}+G^{CAR},$$

and the cross conductance is

(31) $$G_{C}=\frac{\partial I_{R}}{\partial V}{|}_{V=0}=G^{ET}-G^{CAR},$$

where

(32) $$G^{ET}=\frac{e^{2}}{h}\left({\tilde{\Gamma }}_{L1}{\tilde{\Gamma }}_{R1}|G_{d11}^{R}{\left(0\right)|}^{2}+{\tilde{\Gamma }}_{L2}{\tilde{\Gamma }}_{R2}{\left|G_{d22}^{R}\left(0\right)\right|}^{2}\right),$$

(33) $$G^{DAR}=\frac{4e^{2}}{h}{\tilde{\Gamma }}_{L1}{\tilde{\Gamma }}_{L2}{\left|G_{d12}^{R}\left(0\right)\right|}^{2},$$

(34) $$G^{CAR}=\frac{e^{2}}{h}\left({\tilde{\Gamma }}_{L1}{\tilde{\Gamma }}_{R2}+{\tilde{\Gamma }}_{R1}{\tilde{\Gamma }}_{L2}\right){\left|G_{d12}^{R}\left(0\right)\right|}^{2}.$$

It is obvious that all of the three different tunneling processes contribute to the local conductance. Nevertheless, the DAR tunneling process, as expected, has no contribution to the cross conductance. More interestingly, the CAR tunneling process provides a contrary contribution, in comparison with the ET process, to the cross-conductance Equation (31), which is responsible for the negative value of the cross conductance in certain appropriate conditions, as shown in the following section. This opposite role of the CAR can be interpreted in an intuitive way: A hole entering the right lead is physically equivalent to an electron breaking into the QD from the right lead, thus resulting in an opposite current flowing in the right lead. It is important to point out that if the superconducting coupling is switched off ($\Gamma_{s}=0$), there are no DAR and CAR processes, and as a result, the cross conductance reduces to the local conductance.

## 3. Result and Discussion

We suppose that the left and right leads are made from the same material and in the wide band limit, that which is of interest in the present investigation, the ferromagnetism of the leads can be accounted for by the polarization-dependent couplings $\Gamma_{L1}=\Gamma_{R1}=\left(1+p\right)\Gamma$, $\Gamma_{L2}=\Gamma_{R2}=\left(1-p\right)\Gamma$ for the parallel (P) alignment, while $\Gamma_{L1}=\Gamma_{R2}=\left(1+p\right)\Gamma$, $\Gamma_{L2}=\Gamma_{R1}=\left(1-p\right)\Gamma$ for the anti-parallel (AP) alignment. Here, Γ describes the tunneling coupling between the QD and the nonmagnetic leads, which is taken as the energy unit in the following calculations. In addition, *p* (0≤p<1) denotes the polarization strength of the leads. The Kondo temperature in the case of p=0, given by $T_{K}=U\sqrt{D}\exp\left(-\pi /D\right)/2\pi$ with $D=-2U\Gamma /\epsilon_{d}\left(U+\epsilon_{d}\right)$, will be set as another dynamical energy scale of the nonlinear conductance.

In the following, we deal with the three-terminal QD system having a fixed finite Coulomb interaction U=10 at zero temperature and consider the effects of changing the bare dot level $\epsilon_{d}$, the spin polarization *p*, and the proximity strength $\Gamma_{s}$, respectively.

### 3.1. Linear Local and Cross Conductances

**Figure 2:**
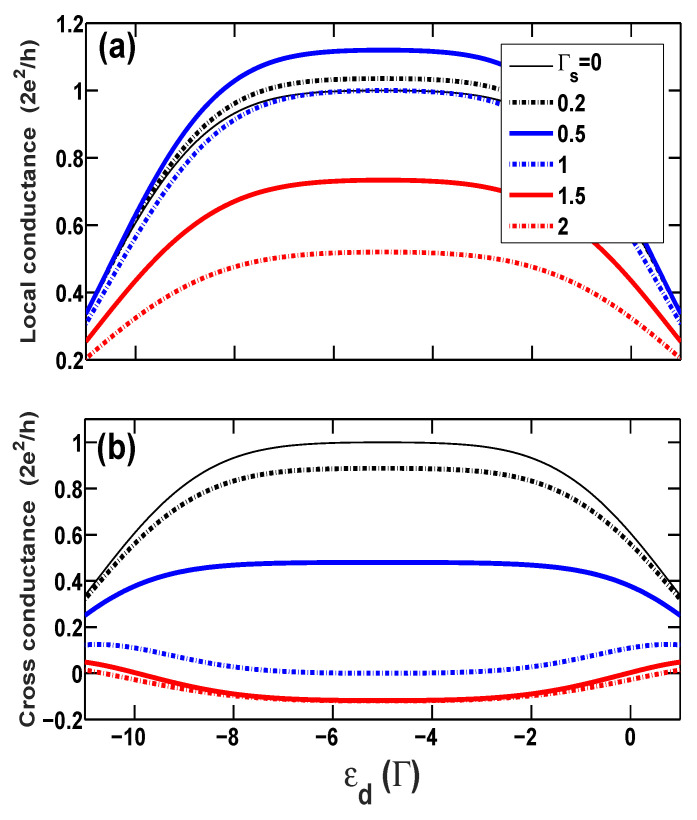
(Color online) (**a**) The local conductance and (**b**) the cross conductance vs. the bare dot level $\epsilon_{d}$ at zero temperature for different proximity-coupling strengths $\Gamma_{s}$ in the case of normal leads, i.e., p=0.

Firstly, we show the calculated linear conductances in Figure 2, including the local conductance $G_{L}$ and the nonlocal cross conductance $G_{C}$ as functions of the bare energy level $\epsilon_{d}$ of the QD at different superconducting coupling strengths, $\Gamma_{s}=0$, 0.2, 0.5, 1.0, 1.5, and 2.0, in the case of no spin-polarization p=0. Without the superconducting coupling $\Gamma_{s}=0$, $G_{L}=G_{C}$ and the linear conductance reaches the unitary limit, $G_{0}$ ($G_{0}\equiv 2e^{2}/h$), as expected in the Kondo regime. With increasing the coupling $\Gamma_{s}$, the local conductance $G_{L}$ raises at the beginning, as seen in Figure 2a, since the AR channel starts to emerge and contribute to the electronic tunneling. A slightly bigger value of conductance, $G_{L}≃1.1G_{0}$, than the unitary limit of conductance of single-particle tunneling is reached at the coupling $\Gamma_{s}=0.5$ in the Kondo regime. On the other hand, it is known that the resonant AR leads to the unitary limit of conductance, $2G_{0}$, of the Cooper pair tunneling in the two-terminal hybrid system, e.g., a normal metal-QD-superconductor system [24]. We can therefore deduce that such a larger value of the conductance is a signature indicating that the tunneling event in the present hybrid system is a mixture of the single-particle and Cooper pair tunnelings. Increasing the coupling $\Gamma_{s}$ further will, however, cause a decrease in the local conductance $G_{L}$. The suppression of $G_{L}$ can be interpreted as follows: An electron coming from the left lead has much higher probability to form the Cooper pair breaking into the superconducting electrode due to the considerable strength of the coupling $\Gamma_{s}>0.5$, and as a result, the ET process is rapidly suppressed. Different from the local conductance, the nonlocal conductance $G_{C}$ decreases from the beginning and even becomes negative if the proximity-coupling is sufficiently strong. The negative cross conductance means that when the left lead is applied with a voltage which is bigger than the right lead, electrons will, instead of entering into the right lead from the QD, tunnel into the QD out of the right lead. Moreover, we find that when the QD leaves the Kondo regime, the cross conductance becomes positive again.

**Figure 3:**
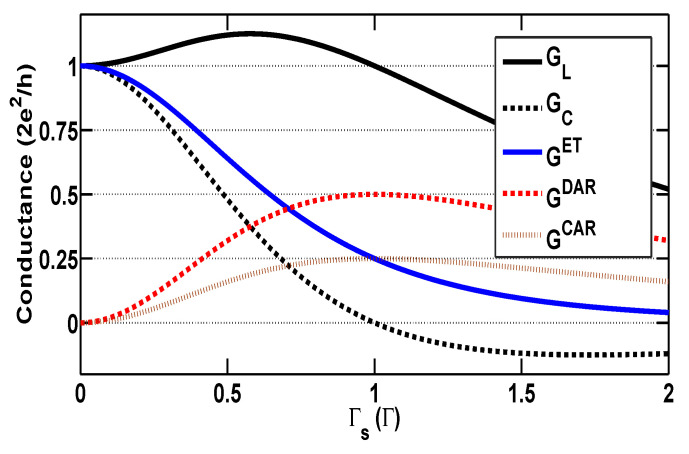
(Color online) The zero temperature local conductance (black-solid line) and the cross conductance (black-dotted line) vs. the proximity coupling $\Gamma_{s}$ for the system with a bare dot level at the particle-hole symmetric point, $\epsilon_{d}=-U/2=-5$ in the case of normal leads (p=0). The three parts of the conductance are also plotted for illustration purposes.

Such effects of $\Gamma_{s}$ are clearly manifested in Figure 3, in which the local and nonlocal conductances, and their three respective parts, $G^{ET}$, $G^{DAR}$, and $G^{CAR}$, are illustrated as functions of the coupling $\Gamma_{s}$ for the specific system which has bare dot level, $\epsilon_{d}=-U/2=-5$. It is observed that a maximum value of the local conductance, $G_{L}=1.125G_{0}$, is arrived at, $\Gamma_{s}=0.58$. After this point of $\Gamma_{s}$, the AR process becomes the predominate tunneling mechanism over the ET process. When the proximity-coupling is equal to the tunnel-coupling, i.e., $\Gamma_{s}=1.0$, a new resonance is reached, originating from interplay between the Kondo effect and AR. Consequently, $G^{DAR}=G_{0}/2$ and $G^{CAR}=G^{ET}=G_{0}/4$, and the local conductance arrive at the unitary value, $G_{L}=G_{0}$ once more. At the same time, the nonlocal conductance completely vanishes, $G_{C}=0$, which indicates no current response in the right lead to the bias voltage applied to the left lead.

**Figure 4:**
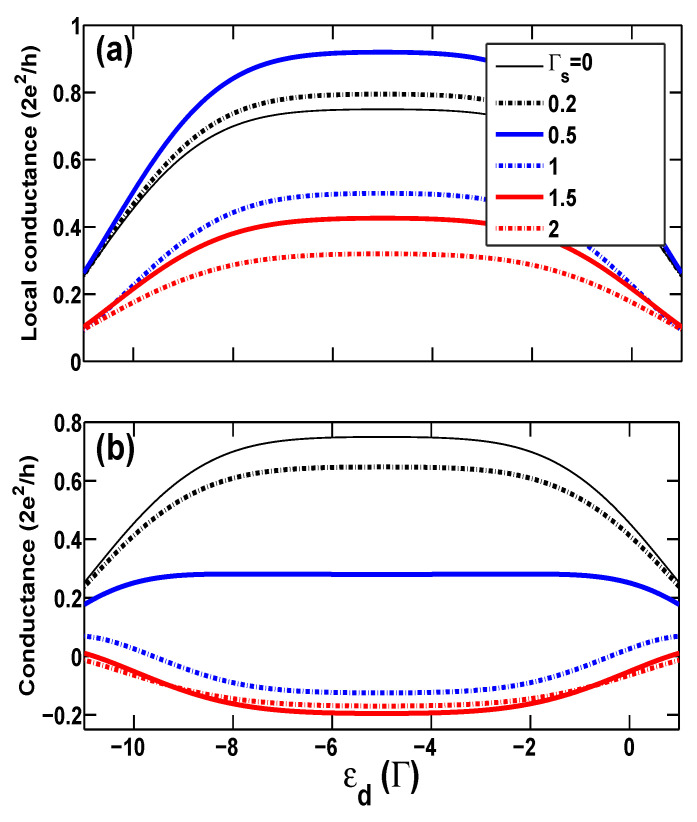
(Colour online) (**a**) The local conductance and (**b**) the cross conductance versus the bare dot level $\epsilon_{d}$ for different proximity-coupling strengths $\Gamma_{s}$ in the AP configuration with p=0.5.

Secondly, in Figure 4, we investigate the cross conductance $G_{C}$ as a function of the bare energy level $\epsilon_{d}$ of the QD at different proximity couplings $\Gamma_{s}$ in the AP configuration with a large spin polarization p=0.5. In the AP configuration, similar with the case of zero spin polarization p=0, electrons with up-spin and down-spin are equally available in the whole system, favoring the formation of the Kondo-correlated state within a wide dot level range centered at $\epsilon_{d}=-U/2=-5$. Meanwhile, since there is no splitting of the renormalized dot levels, $\epsilon_{d}+\lambda_{\sigma }^{2}$, for different spins, the usual tunneling and charging peaks, around $\epsilon_{d}=0$ and −U, respectively, are relatively narrow. The local conductance $G_{L}$ vs. $\epsilon_{d}$ curves show a similar behavior as the case of zero spin polarization even in the presence of superconducting coupling $\Gamma_{s}$. Furthermore, since no spin-flip scattering exists in the tunneling processes, in the AP configuration, the majority-spin (e.g., up-spin) states in the left lead increase but the available up-spin (minority-spin) states in the right lead decrease with increasing spin polarization strength, and as a consequence, the transfer of the majority-spin (up-spin) electrons through the QD is suppressed, such that the local conductance goes down and eventually vanishes at p=1 as expected. On the contrary, the available down-spin states in the right lead increase in the AP configuration, which just facilitates the occurrence of the CAR process [30]. Therefore, one can observe that $G_{C}$ becomes negative in almost the whole region of dot levels, from the mixed-valence regime to the empty orbital regime, even when $\Gamma_{s}<1$, and nearly arrives at a considerably bigger negative value, $G_{C}≃-G_{0}/5$, at the Kondo regime at p=0.5. It is interesting to consider the extreme case of p=1. As mentioned above, in the AP configuration electrons with up-spin and down-spin are identical to each other, preferring the formation of the Kondo-correlated state for all values of *p*. However, since the up-spin states are almost unavailable in the right lead in the case of large polarization, the ET process for the left lead to the right lead is completely damaged (implying an exactly vanishing conductance in the usual QD system), but the CAR process survives here as a unique tunneling mechanism, exclusively making a contribution to electronic tunneling. It is anticipated that in this case, $G^{ET}=G^{DAR}=0$ and $G_{L}=-G_{C}=G^{CAR}=G_{0}/2$ (this is the unitary limit of conductance of the single channel).

**Figure 5:**
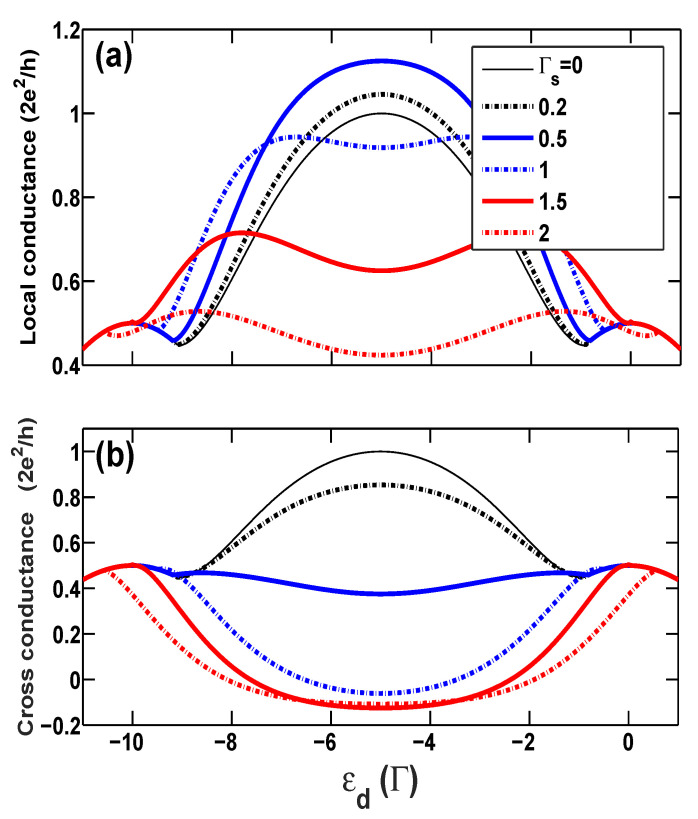
(Color online) (**a**) The local conductance and (**b**) the cross conductance vs. the bare dot level $\epsilon_{d}$ with U=10 at zero temperature for different proximity-coupling strengths $\Gamma_{s}$ in the P configuration with p=0.5.

The situation is quite different in the case of the P configuration, as demonstrated in Figure 5, in which the two conductances are plotted as functions of bare dot level with spin polarization p=0.5. In the P configuration, finite spin polarization splits the dot level for up- and down-spins and thus broadens the usual resonance peaks around $\epsilon_{d}=0$ and $\epsilon_{d}=-U$ [32,33,34,35,36]. On one hand, since minority-spin electrons are still available in the two electrodes to build the Kondo screening correlation to a certain degree, the central Kondo peak can still be reached at the unitary limit $G_{0}$ at the large polarization p=0.5 in the case of $\Gamma_{s}=0$. On the other hand, the number of minority-spin electrons is too small to construct the Kondo-correlated state at p=0.5, and thus Kondo-induced conductance enhancement disappears rapidly when the QD moves away from the particle-hole symmetric point $\epsilon_{d}=-U/2$. These two factors cause the appearance of kinks or splitting peaks in both conductance vs. $\epsilon_{d}$ curves. Besides, it can be observed from Figure 5a that the central Kondo peak in the local conductance is progressively splitting with increasing proximity coupling $\Gamma_{s}\geq 1.0$ in this P configuration. Furthermore, a decrease in minority-spin states in both leads in the P configuration hinders the emergence of AR processes, which leads to weakly negative cross conductance in the Kondo regime, e.g., $G_{C}\geq -0.1G_{0}$, and even causes CAR to totally vanish, thus $G_{C}≃G_{L}$ at the two usual resonance peaks, as shown in Figure 5b. This states that strong ferromagnetism destroys proximitized superconductivity in this three-terminal hybrid nanosystem.

### 3.2. Nonlinear Local and Cross Conductances

Now, we turn to the investigation of nonlinear tunneling, since the nonlinear differential conductance $dI_{L}/dV$ is believed to be a very useful tool in experiments aimed at detecting the formation of the Kondo-correlated state due to its proportionality to the transmission spectrum, supposing that the total transmission is unchanged subject to the external bias voltage. In the present three-terminal hybrid device, one can define the local and cross differential conductances, $g_{L}=\partial I_{L}/\partial V$ and $g_{C}=\partial I_{R}/\partial V$, if the bias voltage *V* is applied to the left lead and while the superconducting and the right leads are kept grounded. From the Equations (26)–(29), we can obtain that the two diffenertial conductances are both proportional to the normal transmission spectrum $T_{N}\left(\omega \right)$ and the AR spectrum $T_{A}\left(\omega \right)$ at ω=V at zero temperature, $g_{L}\propto T_{N}\left(V\right)+aT_{A}\left(V\right)$ and $g_{C}\propto T_{N}\left(V\right)-bT_{A}\left(V\right)$ (*a* and *b* are constants).

**Figure 6:**
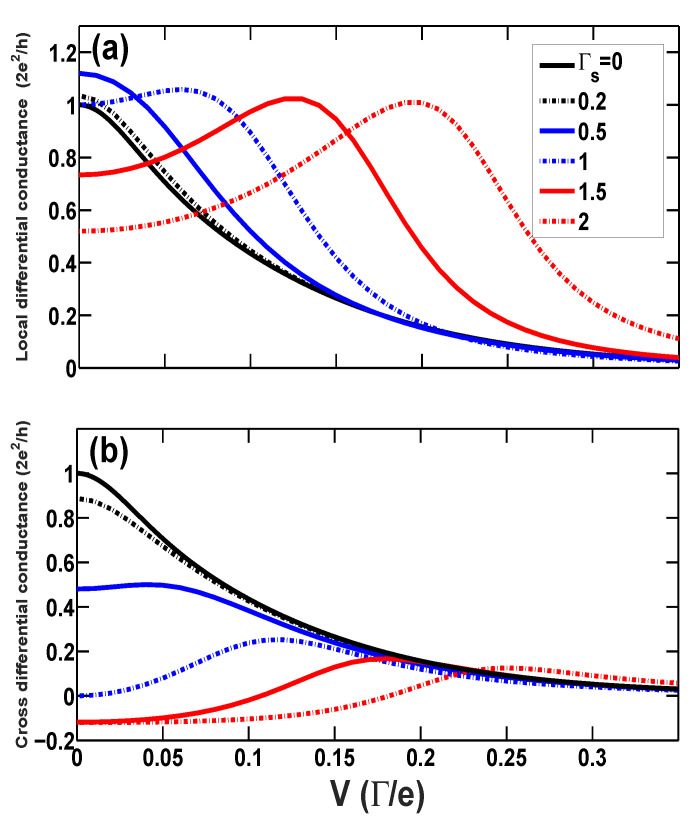
(Color online) The zero-temperature local (**a**) and cross (**b**) differential conductances vs. bias voltage *V* for various couplings $\Gamma_{s}$ for the system with bare dot level $\epsilon_{d}=-5$ and U=10 in the case of normal leads (p=0).

Figure 6 shows the local and cross differential conductances as functions of bias voltage at various proximity couplings $\Gamma_{s}$ for the system with a single dot level $\epsilon_{d}=-5$ ($T_{K}≃0.03$) at the Kondo regime. These curves for weak proximity coupling $\Gamma_{s}<1.0$ present a single zero-bias anomaly, which is the signature of the Kondo effect. Nevertheless, there appears non-zero-bias peak with increasing proximity coupling $\Gamma_{s}\geq 1.0$. It is announced that the Kondo correlation enhances not only the normal ET, but also the AR; nonetheless. the increasing superconducting proximity coupling induces splitting of the Kondo peaks in the normal transmission spectrum as well as the AR spectrum. This peak splitting is the reason that the three parts of the linear conductance are all suppressed when $\Gamma_{s}>1.0$, as shown in Figure 3. Finally, one can observe that the negative cross differential conductance becomes positive in the case of large bias voltage. External bias voltage plays a role in dissipation so as to destroy not only the Kondo correlation but the negative nonlocal current response as well.

## 4. Conclusions

We have theoretically investigated the subgap transport properties of a hybrid nanosystem consisting of an interacting QD connected to one superconducting lead and two ferromagnetic leads. On the basis of the finite-*U* slave boson mean field approach and the NGF method, we find markedly rich transport features ascribed to the competition among the Kondo correlation, superconducting proximity effect, and spin polarization of electrodes. In the case of weak superconducting proximity coupling, the Kondo-correlated state can still be built, leading to a single zero-bias peak in the voltage-dependent differential conductance. However, the peak height drops down gradually with increasing $\Gamma_{s}$, and when $\Gamma_{s}\geq 1.0$, a non-zero peak appears. Such strong proximity coupling induces linear cross conductance which is negative in the Kondo region. Spin polarization can further enhance the opposite current response in the right lead (more negative cross conductance) in the AP configuration, because such a configuration is advantageous to the emergence of CAR. In contrast, in the P configuration, the rising spin polarization *p* blocks the CAR process and also splits the Kondo peak, such that the linear local conductance exhibits four peaks when $\Gamma_{s}\geq 1.0$, and the linear cross conductance reduces to the normal positive conductance more rapidly.

## Appendix A

In this appendix, we briefly show how to obtain the effective Hamiltonian Equation (7). Within the formulation of the finite-*U* slave boson approach, the original QD electron operators are replaced by the new varibales as follows: $n_{1}n_{2}\to d^{†}d$, $c_{d\sigma }\to z_{\sigma }f_{d\sigma }$, and $c_{d\sigma }^{†}c_{d\sigma }\to f_{d\sigma }^{†}f_{d\sigma }$. The additional degrees of freedom simplify the Coulomb interaction $Un_{1}n_{2}$ as $Ud^{†}d$, but introduce vectors not describing physically real states. Then, two constraint conditions have to be imposed to eliminate the unphysical part of the enlarged Hilbert space |Φ〉

(A1) $$\begin{array}{cc} \; & (\sum_{\sigma }p_{\sigma }^{†}p_{\sigma }+e^{†}e+d^{†}d-1)|\Phi \rangle =0,\\ \; & (f_{d\sigma }^{†}f_{d\sigma }-p_{\sigma }^{†}p_{\sigma }-d^{†}d)|\Phi \rangle =0. \end{array}$$

Therefore, the subspace of the enlarged Hilbert space defined by Equation (A1) is equivalent to the original Hilbert space. Applying Dirac’s formulation of constrained dynamics, one should then introduce two *q*-number Lagrange multipliers $\lambda^{1}$ and $\lambda_{\sigma }^{2}$, corresponding to the two constraints in Equation (A1), to the Heisenberg equation of motion with respect to the effective Hamiltonian

(A2) $$\tilde{H}\equiv H+\lambda^{1}\left(\sum_{\sigma }p_{\sigma }^{†}p_{\sigma }+e^{†}e+d^{†}d-1\right)+\sum_{\sigma }\lambda_{\sigma }^{2}\left(f_{d\sigma }^{†}f_{d\sigma }-p_{\sigma }^{†}p_{\sigma }-d^{†}d\right),$$

and consequently any dynamical observable $\hat{A}$ satisfies the standard equation of motion as a state equation

(A3) $$i\hbar \frac{d\hat{A}}{dt}|\Phi \rangle =\left[\hat{A},\tilde{H}\right]|\Phi \rangle .$$

The next key point of the SBMF approach is to replace all the slave-boson operators and the Lagrange multipliers by their average values according to nonequilibrium steady states (NESS), which can be still expressed in this paper by *e*, $p_{\sigma }$, *d*, $\lambda_{1}$, and $\lambda_{\sigma }^{2}$. As a result, we can indeed obtain the mean-field expression of the effective Hamiltonian Equation (7) (please note that we still use the notation $c_{d\sigma }$ instead of $f_{d\sigma }$ in the effective Hamiltonian in the main text for the sake of convenience).

## Appendix B

It is easily noticed that there are seven averages in total to be determined within the SBMF approach, and the constraints provide three conditions: Equations (13) and (14). Hence, four more conditions are necessary. Originally, Kotliar and Ruckenstein [46] derived them by the saddle-point approximation for the equilibrium free energy, but this approach cannot be applied in this paper, since we are dealing with the NESS. Instead, herein, we can derive those conditions from the equations of motion of the four slave boson fields according to Equation (A3). For instance, the empty boson field *e* obeys the equation of motion:

(A4) $$i\hbar \frac{de}{dt}=\Gamma_{s}\left(\left[e,z_{1}^{†}z_{2}^{†}\right]c_{d1}^{†}c_{d2}^{†}+\left[e,z_{1}z_{2}\right]c_{d2}c_{d1}\right)+\sum_{\eta k\sigma }V_{\eta k}\left(c_{\eta k\sigma }^{†}c_{d\sigma }\left[e,z_{\sigma }\right]+c_{d\sigma }^{†}c_{\eta k\sigma }\left[e,z_{\sigma }^{†}\right]\right)+\lambda^{1}e.$$

By replacing the slave-boson operators and Lagrange multipliers to their mean values and evaluating NESS averages of the fermionic operators, one obtains, in the condition of steady state,

(A5) $$i\hbar \langle \frac{de}{dt}\rangle =\Gamma_{s}\left(\frac{\partial z_{1}^{*}z_{2}^{*}}{\partial e^{*}}\langle c_{d1}^{†}c_{d2}^{†}\rangle +\frac{\partial z_{1}z_{2}}{\partial e^{*}}\langle c_{d2}c_{d1}\rangle \right)+\sum_{\eta k\sigma }V_{\eta k}\left(\frac{\partial z_{\sigma }}{\partial e^{*}}\langle c_{\eta k\sigma }^{†}c_{d\sigma }\rangle +\frac{\partial z_{\sigma }^{*}}{\partial e^{*}}\langle c_{d\sigma }^{†}c_{\eta k\sigma }\rangle \right)+\lambda^{1}e=0.$$

Here the commutators of the boson fields are evaluated as $\left[e,z_{\sigma }\right]=\partial z_{\sigma }/\partial e^{†}$ and $\left[e,z_{1}z_{2}\right]=\partial z_{1}z_{2}/\partial e^{†}$, according to the Dirac’s quantum algebraic scheme.

Now, we turn to discuss the averages of fermionic operators which are evaluated at a NESS with respect to the fermionic part of the effective Hamiltonian Equation (7). Since we discuss quantum transport through a nanodevice in this paper, a NESS can be defined as being when all electrodes are at equilibrium with their own temperatures $1/\beta_{\eta }$ and chemical potentials $\mu_{\eta }$, and thus the statistical operator can be introduced as

(A6) $$\varrho_{0}=\frac{1}{\Xi }e^{-\beta_{L}\left(H_{L}-\mu_{L}N_{L}\right)}e^{-\beta_{R}\left(H_{R}-\mu_{R}N_{R}\right)}.$$

In the above, Ξ is the normalization constant, $H_{\eta }$ and $N_{\eta }$ are the Hamiltonian and the total number of particles in the lead η, respectively. According to the nonequilibrium statistical theory, the average of an observable $\hat{A}$ with respect to such a NESS can then be evaluated as $\langle \hat{A}\rangle =Tr\left(\hat{A}\varrho_{0}\right)$. Thereby, we can evaluate the averages of fermionic operators with the help of the NGF technique, for example, the operators of occupation number on the dot, $K_{1}$ and $K_{2}$ (Equations (15) and (16)), and the order parameter *R* in Equation (17). Moreover, we can define

(A7) $$Q_{1\eta }=\sum_{k}V_{\eta k}\langle c_{d1}^{†}c_{\eta k1}\rangle =\sum_{k}V_{\eta k}\int \frac{d\omega }{2\pi i}{\langle \langle c_{\eta k1};c_{d1}^{†}\rangle \rangle }^{<}\left(\omega \right)=\sum_{k}V_{\eta k}\int \frac{d\omega }{2\pi i}G_{\eta k,d;11}^{<}\left(\omega \right),$$

and

(A8) $$Q_{2\eta }=\sum_{k}V_{\eta k}\langle c_{\eta k2}^{†}c_{d2}\rangle =\sum_{k}V_{\eta k}\int \frac{d\omega }{2\pi i}{\langle \langle c_{\eta k2}^{†};c_{d2}\rangle \rangle }^{<}\left(\omega \right)=\sum_{k}V_{\eta k}\int \frac{d\omega }{2\pi i}G_{\eta k,d;22}^{<}\left(\omega \right).$$

Here, the hybrid NGFs, $G_{\eta k,d}^{<}\left(\omega \right)$ are the matrix elements of the 2×2 hybrid contour-order GF matrix $G_{\eta k,d}^{\gamma }\left(\omega \right)={\langle \langle L_{\eta k};\phi^{†}\rangle \rangle }^{\gamma }$ (γ=R,A,<,>) with the Nambu representation in the lead $L_{\eta k}={\left(c_{\eta k1},c_{\eta k2}^{†}\right)}^{T}$. Applying the Langreth theorem, we obtain

(A9) $$G_{\eta k,d}^{<}\left(\omega \right)=g_{\eta k}^{R}\left(\omega \right){\hat{V}}_{\eta k}G_{d}^{<}\left(\omega \right)+g_{\eta k}^{<}\left(\omega \right){\hat{V}}_{\eta k}G_{d}^{A}\left(\omega \right),$$

where $g_{\eta k}^{\gamma }$ is the decoupled NGF of the lead η defined as $g_{\eta k}^{\gamma }\left(\omega \right)={\langle \langle L_{\eta k};L_{\eta k}^{†}\rangle \rangle }^{\gamma }$ and

(A10) $${\hat{V}}_{\eta k}=\left(\begin{array}{cc} z_{1}V_{\eta k} & 0\\ 0 & -z_{2}V_{\eta k}^{*} \end{array}\right).$$

Subsequently, we have

(A11) $$\begin{array}{cc} \sum_{k}{\hat{V}}_{\eta k}G_{\eta k,d}^{<}\left(\omega \right)= & \sum_{k}{\hat{V}}_{\eta k}g_{\eta k}^{R}\left(\omega \right){\hat{V}}_{\eta k}G_{d}^{<}\left(\omega \right)+{\hat{V}}_{\eta k}g_{\eta k}^{<}\left(\omega \right){\hat{V}}_{\eta k}G_{d}^{A}\left(\omega \right),\\ = & \Sigma_{\eta }^{R}\left(\omega \right)G_{d}^{<}\left(\omega \right)+\Sigma_{\eta }^{<}\left(\omega \right)G_{d}^{A}\left(\omega \right), \end{array}$$

with

(A12) $$\Sigma_{\eta }^{\gamma }\left(\omega \right)=\sum_{k}{\hat{V}}_{\eta k}^{*}g_{\eta k}^{\gamma }\left(\omega \right){\hat{V}}_{\eta k}=\left(\begin{array}{cc} \sum_{k}{\left|V_{\eta k}\right|}^{2}g_{\eta k;11}^{\gamma }\left(\omega \right) & 0\\ 0 & \sum_{k}{\left|V_{\eta k}\right|}^{2}g_{\eta k;22}^{\gamma }\left(\omega \right) \end{array}\right).$$

Noticing the retarded and advanced self energies in the wide band limit,

(A13) $$\Sigma_{\eta }^{R(A)}=\pm \frac{i}{2}\left(\begin{array}{cc} {\tilde{\Gamma }}_{\eta 1} & 0\\ 0 & {\tilde{\Gamma }}_{\eta 2} \end{array}\right),$$

and Equations (21)–(24), we can further obtain an explicit expression of Equation (A11) and insert this expression into Equations (A7) and (A8) to yield Equations (18) and (19). Finally, we obtain the self-consistent equation Equation (9).

Similarly, from the equations of motion of $p_{\sigma }$ and *d*,

(A14) $$\begin{array}{cc} i\hbar \frac{dp_{\sigma }}{dt}= & \Gamma_{s}\left(\left[p_{\sigma },z_{1}^{†}z_{2}^{†}\right]c_{d1}^{†}c_{d2}^{†}+\left[p_{\sigma },z_{1}z_{2}\right]c_{d2}c_{d1}\right)+\sum_{\eta k\sigma^{\prime }}V_{\eta k}\left(c_{\eta k\sigma^{\prime }}^{†}c_{d\sigma^{\prime }}\left[p_{\sigma },z_{\sigma }^{\prime }\right]+c_{d\sigma^{\prime }}^{†}c_{\eta k\sigma^{\prime }}\left[p_{\sigma },z_{\sigma }^{\prime †}\right]\right)\\ \; & +\left(\lambda^{1}-\lambda_{\sigma }^{2}\right)p_{\sigma }, \end{array}$$

(A15) $$\begin{array}{cc} i\hbar \frac{dd}{dt}= & \Gamma_{s}\left(\left[d,z_{1}^{†}z_{2}^{†}\right]c_{d1}^{†}c_{d2}^{†}+\left[d,z_{1}z_{2}\right]c_{d2}c_{d1}\right)+\sum_{\eta k\sigma }V_{\eta k}\left(c_{\eta k\sigma }^{†}c_{d\sigma }\left[d,z_{\sigma }\right]+c_{d\sigma }^{†}c_{\eta k\sigma }\left[d,z_{\sigma }^{†}\right]\right)\\ + & (U+\lambda^{1}-\Sigma_{\sigma }\lambda_{\sigma }^{2})d, \end{array}$$

we can get self-consistent equations, Equations (10)–(12).
